# Cutaneous melanoma, prostate-specific antigen testing and the subsequent risk of prostate cancer diagnosis: a prospective analysis of the 45 and Up Study

**DOI:** 10.1038/s41416-022-02027-7

**Published:** 2022-11-01

**Authors:** Sam Egger, David P. Smith, Manish I. Patel, Michael G. Kimlin, Bruce K. Armstrong, Visalini Nair-Shalliker

**Affiliations:** 1 The Daffodil Centre, The University of Sydney, a Joint Venture with Cancer Council New South Wales, Sydney, NSW Australia; 2 School of Public Health and Preventative Medicine, Monash University, Melbourne, VIC Australia; 3 Specialty of Surgery, Sydney Medical School, The University of Sydney and Department of Urology, Westmead Hospital, Sydney, NSW Australia; 4 School of Biomedical Sciences, Queensland University of Technology, Brisbane, QLD Australia; 5 School of Population and Global Health, University of Western Australia, Perth, WA Australia; 6 Faculty of Medicine and Health Sciences, Macquarie University, Sydney, NSW Australia

**Keywords:** Risk factors, Cancer epidemiology

## Abstract

**Background:**

The association between cutaneous melanoma and subsequent risk of prostate cancer (PC) was examined in a large population-based cohort study.

**Methods:**

Male participants in the Sax Institute’s 45 and Up Study (Australia) were recruited between 2006 and 2009. Questionnaire data and linked administrative health data from the Centre for Health Record Linkage and Services Australia identified melanomas diagnosed between 1/1/1994 and 12 months before Study recruitment (i.e., between 2005 and 2008), incident PCs, primary healthcare utilisation and prostate-specific antigen (PSA) tests. Men were excluded from the current analyses if they had a recorded PC or other cancer diagnosis other than melanoma and non-melanoma skin cancer prior to recruitment. Multivariable Cox regression was used to estimate hazard ratios (HRs) adjusting for PSA-testing frequency before PC diagnosis.

**Results:**

Of 96,548 eligible men, 1899 were diagnosed with melanoma during the melanoma diagnosis period and 3677 incident PC diagnosed during follow-up (latest date 31/12/2013). Men with melanoma diagnosis had increased risk of a subsequent PC diagnoses (vs. no melanoma; fully adjusted HR = 1.32; 95% CI: 1.09–1.60). There was weak evidence of higher risks of a subsequent PC diagnosis for men diagnosed with more than one melanoma compared to men diagnosed with only one melanoma (*p* = 0.077), and if first melanoma diagnosis was 10 to 15 years before Study recruitment (fully adjusted HR = 2.05; 95% CI [1.35, 3.12]).

**Conclusion:**

Melanoma diagnosis was associated with increased risk of subsequent PC diagnosis, after adjusting for PSA testing and primary healthcare utilisation. While our ability to adjust for PC screening reduced risk of detection bias, we acknowledge that residual confounding from increased medical surveillance after melanoma diagnoses cannot be entirely ruled out.

## Introduction

Prostate cancer (PC) was the most diagnosed cancer among Australian men in 2020, comprising about 25% of all newly diagnosed cancers in men [1]. In the same year, cutaneous melanoma (referred to here as melanoma) was the third most diagnosed cancer among Australian men, comprising about 10% of all newly diagnosed cancers among them [1]. In comparison with other countries, Australia had the highest and 15th highest incidence rates of melanoma and PC, respectively, in 2020 [2]. Highly correlated incidences of PC and melanoma have also been observed in other regions of the world such as North America and Europe and perhaps in other world regions [2].

Solar ultraviolet radiation (UVR) is an established risk factor for melanoma [3]. In Australia, over 95% of melanoma cases are thought to be attributable to UVR [3, 4], compared to about 75% worldwide [3]. UVR may also be associated with PC risk, although the nature of this relationship is unclear. Several studies have found that UVR reduces the risk of PC [5], although two observational studies conducted in high UV environments—Australia and Singapore—suggest an association between high levels of UVR and increased PC risk. More evidence is needed to substantiate these findings [6, 7].

Few studies have investigated the association between melanoma and subsequent risk of various internal cancers such as prostate cancer [8–10]. A recent systematic review of 17 studies, found that men diagnosed with melanoma were at subsequent higher risk of PC diagnosis than men in general [11]. Its findings, however, do not make clear whether melanoma is a true risk factor for PC or whether detection bias due to increased medical surveillance following a melanoma diagnosis is responsible. As a result of increased interaction with the health system it is possible that more asymptomatic PC is being detected due to more prostate-specific antigen (PSA) testing. Consistent with latter of these two theories is the fact that all but one study included in the review found that PCs were most likely to be detected in the first year following the diagnosis of melanoma (a period when medical surveillance is expected to be high). Moreover, none of the 17 studies included in the review adjusted for potential confounders related to medical surveillance. Improving our understanding of these underlying factors is important for knowledge of PC considering there are no known modifiable PC risk factors.

Given the absence of studies that have accounted for potential confounding from medical surveillance when examining the risk of PC after a melanoma diagnosis, the current study aims to fill this void. More specifically, we aim to estimate the effect of melanoma diagnosis on the subsequent risk of PC diagnosis while accounting for frequency of prostate-specific antigen (PSA) testing and general practitioner (GP) consultations. Secondary aims are to assess whether PC risk differs according to clinical characteristics of melanoma.

## Methods

### Study population

The Sax Institute’s 45 and Up Study is Australia’s largest ongoing study of health and ageing, its methods are described in detail elsewhere [12, 13]. Briefly, the 45 and Up Study is a cohort study of 267,153 people aged 45 and over at baseline, randomly sampled from the population of New South Wales (NSW), Australia, using the Services Australia (formerly the Australian Government Department of Human Services) Medicare enrolment database (which contains records for all citizens and permanent residents of Australia). It has oversampled people aged 80+ years, from regional and rural areas, and with higher average incomes compared to the general population. At the time of record linkage, data for 123, 747 male participants were available for analysis.

The 45 and Up Study was approved by the University of NSW Human Research Ethics Committee, while approval for this analysis of PC risk factors was given by the NSW Population and Health Services Research Ethics Committee (HREC/14/CIPHS/54). The use of Services Australia’s Medicare Benefits Schedule (MBS) and Pharmaceutical Benefits Scheme (PBS) data was approved by the Australian Department of Health’s Departmental Ethics Committee.

### Data sources

Participants completed a postal baseline questionnaire between January 2006 and December 2009. Baseline questionnaire data from study participants were probabilistically linked to several population health and administrative databases including: (1) the NSW Cancer Registry (NSWCR) database for all cancers other than non-melanoma skin cancer diagnosed between January 1994 and December 2013; (2) the Register of Births, Deaths, and Marriages database containing notifications of all deaths in NSW occurring between January 2006 and December 2015; (3) the Cause of Death Unit Record File (CODURF) containing cause of death information for all deaths in NSW occurring between January 2006 and December 2015; (4) the Medicare Benefits Schedule (MBS) data containing information on all medical, procedural and diagnostic procedures for which Services Australia paid a subsidy occurring between December 2016 and either June 2004 for participants recruited before April 2008, or September 2005 for participants recruited before April 2008 and (5) the NSW Admitted Patient Data Collection (APDC) containing information on all public and private hospital admissions in NSW between July 2001 and June 2016. Data linkage was performed by the Centre for Health Record Linkage (CHeReL) using the open-source probabilistic record linkage software Choice Maker [14]. The linkage process has been shown to be highly accurate with false-positive and false-negative rates of less than 0.4% [15].

### Exposure and outcome variables

**Fig. 1:**
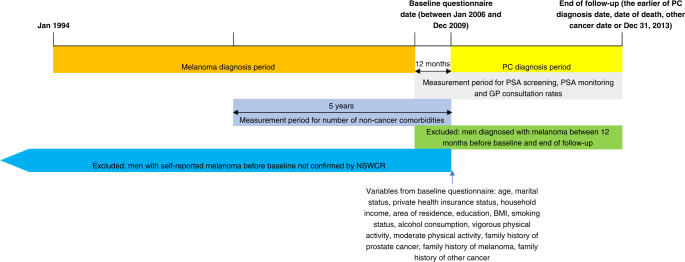
Time periods for measurement of study variables and exclusion criteria related to melanoma. Not to scale.

The primary exposure in these analyses was diagnosis of melanoma during a specified time interval, referred to hereafter as the “melanoma diagnosis period”. For each participant, the melanoma diagnosis period was from 1st January 1994, the earliest date of diagnosis for linked NSWCR records, to 12 months before the date on which the participant completed their baseline questionnaire (Fig. 1). Incident melanoma diagnoses were identified by International Classification of Disease-10 (ICD-10) codes C43 and D03 in the NSWCR linked data. Other melanoma variables were: number of melanoma diagnoses during the melanoma diagnosis period (0, 1, 2+), invasiveness of first melanoma during the melanoma diagnosis period (no melanoma, invasive, in situ), Breslow thickness of first melanoma (no melanoma, 0.01–1.00 mm, 1.01–2.00 mm, >2.00 mm), stage of first melanoma (no melanoma, localised, regional, distant, unknown), site of first melanoma (no melanoma, head and neck, trunk, upper/lower limbs, unspecified/overlapping), time since first melanoma diagnosis (no melanoma, >1–5 years, >5 to 10 years, >10 to 15 years). The primary outcome was the first PC diagnosis (identified through the NSWCR linked data as ICD-10 code C61) after baseline, and before the earlier of December 31, 2013, date of death or date of diagnosis of a cancer other than PC, the “PC diagnosis period” (Fig. 1).

### Baseline variables

The baseline 45 and Up Study questionnaire collected information on participants’ socio-demographic factors, health behaviours, anthropometric measurements, medical and surgical history, physical activity and family history of selected diseases. From this information, baseline variables were constructed for use as covariates in regression analyses, including: age (treated continuously), marital status (single, widowed/divorced/separated, married/living with partner, no response), private health insurance status (none, healthcare concession card/private health insurance, unknown), household income (less than $19,999, $20,000–$39,999 per year, $40,000–$69,999 per year, $70,000 or more per year, unknown), place of residence identified by the residential postcode and classified according to the Accessibility Remoteness Index of Australia (ARIA+) (major cities, inner regional, outer regional, remote, very remote, unknown), highest level of education/qualification (no school certificate or other qualification, school/intermediate certificate/trade or apprenticeship, certificate or diploma, university degree or higher, unknown), body mass index (BMI) (≤18, 18–24.9, 25–29.9, 30–34.9, 35–39.9, ≥40 kg/m^2^, unknown), smoking status (never smoker, ex-smoker, current-smoker, unknown), alcohol consumption (non-drinkers, 1 to <15, ≥15 drinks/week, unknown), vigorous physical activity (0, >0 to <5, 5 to <10 ≥ 10 hours/week, unknown), moderate physical activity (0, >0 to <5, 5 to <10, 10 to <15, ≥15 hours/week, unknown), family history of PC (no/unknown, yes), family history of melanoma (no/unknown, yes) and family history of other cancer (no/unknown, yes).

### Medical surveillance and comorbidity variables

MBS linked data was used to quantify general practitioner (GP) consultations (Medicare item numbers 3, 23, 36, 44, 20, 35, 43, 51, 5000, 5020, 5040, 5060, 5010, 5028, 5049, 5067, 4, 24, 37, 47, 585, 599, 597, 5003, 5023, 5043, 5063, 701, 703, 705, 707, 715, 700, 702, 712, 717), PSA-screening tests (Medicare item number 66655, which can be claimed only once every 12 months and is generally used to test men with no previous history of high PSA or prostatic disease) and PSA monitoring tests (Medicare item numbers 66656, 66659 and 66660, are used when monitoring men with previously high PSA and/or prostatic disease). From this data, three “medical surveillance” covariates were constructed measuring – for each participant from 12 months before baseline to the end of the PC diagnosis period (Fig. 1)—the number of GP visits per year (0 to <2, 2 to <4, 4 to <6, 6 to <8, 8 to <10, 10+ visits/year), the number of PSA-screening tests per 5 years (0, >0 to 1, >1 to 2, >2 to 3, >3 to 4, >4 tests/5 years) and the number of PSA monitoring tests per 5 years (0, >0 to 1, >1 to 2, >2 to 3, >3 to 4, >4 tests/5 years). The Charlson’s comorbidity index for the 5 years prior to baseline was calculated from in-patient records from the APDC [16]. The index was then used to construct a covariate representing the number of non-cancer comorbidities in the 5 years prior to baseline (0, 1, 2+).

### Statistical methods

Only male participants of the 45 and Up study were included in this analysis (*n* = 123,747). Participants were excluded if they had record linkage or data errors (*n* = 51), held a Department of Veterans Affairs (DVA) healthcare card (*n* = 8739; MBS data do not capture many of the services and procedures provided to DVA cardholders), had a PC diagnosis (*n* = 7192) or another cancer diagnosis other than melanoma and non-melanoma skin cancer prior to baseline (*n* = 4197; ascertained from the NSWCR from 1994 onwards or self-reported in the baseline questionnaire), reported having a melanoma before baseline that could not be confirmed by the NSWCR (*n* = 4288) (because melanoma is known to be self-reported inaccurately [17]), or were diagnosed with melanoma between 12 months before baseline and the end of the PC diagnosis period (to separate the melanoma and PC diagnosis periods thereby removing the potential for confounding from short-term increased medical surveillance after a melanoma diagnosis) (*n* = 2874) (Fig. 1). The analysis dataset consisted of 96,548 men (Supplementary Table 1).

Cox proportional hazard regression analyses with age as the underlying time variable were used to estimate hazard ratios (HRs) and 95% confidence intervals (95% CIs) measuring the associations between melanoma exposure covariates and PC diagnosis [18]. In these analyses, the endpoint was PC diagnosis, or with censoring on the earlier of December 31, 2013, date of death or the diagnosis date of a cancer other than PC. “Fully adjusted” regression models (with hazard ratios denoted as HR_4_) were adjusted for age (as underlying time variable), marital status (stratified), private health insurance status, household income, area of residence, education (stratified), BMI, smoking status, alcohol consumption, vigorous physical activity, moderate physical activity, family history of prostate cancer (stratified), family history of melanoma, family history of other cancer, number of non-cancer comorbidities in past 5 years, number of GP visits and number of PSA screening and monitoring tests at various levels, with specific covariates included in the different models listed below the result tables. Tests of the proportional hazards assumption were performed for all Cox models using Stata’s “esatat phtest” command (which tests the slopes of Schoenfield’s residuals against zero). Where significant violations were detected for specific confounders, “stratified Cox models” were used to adjust for those confounders [19]. No violations of the proportional hazards assumptions were identified for any melanoma exposure covariates; hence no remedial action was needed for these variables.

In the main analyses, Cox regression models were adjusted for rate of PSA screening tests but were not adjusted for rate of PSA monitoring tests. This is because the PSA monitoring test is recommended to be used only for men with a previously high PSA and/or prostatic disease and adjusting for tests performed in response to signs of cancer can produce biased results [20]. Nonetheless, in sensitivity analyses we repeated the main analysis additionally adjusting for rate of PSA monitoring tests per 5 years (“over-adjusted” models with hazard ratios denoted as HR_5_). In additional sensitivity analysis we looked at the association between PC risk and the characteristics of the most recent melanoma diagnosed (as opposed to first melanoma diagnosed) during the melanoma diagnosis period.

## Results

Of the 96,548 included men, 1899 (2.0%) were diagnosed with one or more melanomas during the melanoma diagnosis period, of which 280 (14.7%) had their first melanoma diagnosis >10 to 15 years prior to baseline (Table 1). Most of the first melanomas were localised (*n* = 1237; 65.1%), invasive (*n* = 1392; 73.3%) and about half had Breslow thickness ≤1.00 mm (*n* = 942; 49.6%). Compared to men who did not have a melanoma diagnosis, melanoma cases tended to be older (mean age 67.8 vs. 62.0 years), less likely to have a household income greater than $70,000 (24.4% vs. 31.1%) and more likely to have family history of melanoma (14.3% vs. 7.1%). Men with a melanoma diagnosis had on average 3.2 GP visits per year compared to 2.6 GP visits per year for men with no melanoma diagnosis (Table 2). Both men with and without a melanoma diagnosis had on average 1.3 PSA-screening tests every 5 years.

**Table 1:** Characteristics of study participants by first melanoma characteristics.

		Melanoma						
Characteristic	No. of melanoma	All first melanomas	>1 Melanomas	Invasive^a^	Breslow^a^ ≤1.00 mm	Localised^a^	Trunk region^a^	>10 years since diagnosis^a^
Total, *n* (%)	94,649 (98.0)	1899 (2.0)	71 (0.1)	1392 (1.4)	942 (1.0)	1237 (1.3)	809 (0.8)	280 (0.3)
Age: mean (SE)	62.0 (0.03)	67.8 (0.24)	71.5 (1.25)	67.6 (0.28)	67.5 (0.29)	66.8 (0.34)	67.3 (0.36)	68.3 (0.63)
45–64 years, *n* (%)	60,382 (63.8)	731 (38.5)	15 (21.1)	546 (39.2)	388 (41.2)	487 (39.4)	319 (39.4)	106 (37.9)
65–79 years, *n* (%)	27,139 (28.7)	893 (47.0)	42 (59.2)	644 (46.3)	441 (46.8)	574 (46.4)	381 (47.1)	125 (44.6)
80+ years, *n* (%)	7128 (7.5)	275 (14.5)	14 (19.7)	202 (14.5)	113 (12.0)	176 (14.2)	109 (13.5)	49 (17.5)
Married/living with partner, *n* (%)	75,945 (81.0)	1567 (83.2)	54 (78.3)	1141 (82.7)	789 (84.7)	1030 (83.9)	670 (84.1)	235 (84.8)
Private health insurance, *n* (%)	60,419 (63.8)	1305 (68.7)	43 (60.6)	941 (67.6)	648 (68.8)	848 (68.6)	537 (66.4)	198 (70.7)
Household income $70,000+, *n* (%)	28,383 (31.1)	445 (24.4)	13 (20.6)	313 (23.4)	219 (24.3)	283 (23.8)	183 (23.5)	60 (22.1)
Residing in major city, *n* (%)	50,092 (53.7)	971 (51.7)	41 (57.7)	702 (51.1)	466 (50.2)	617 (50.7)	416 (52.1)	137 (49.3)
University degree, *n* (%)	24,436 (26.3)	429 (23.0)	11 (15.9)	306 (22.4)	212 (22.8)	272 (22.4)	164 (20.7)	61 (21.9)
Body mass index: mean (SE)	27.3 (0.01)	27.3 (0.10)	27.3 (0.54)	27.3 (0.11)	27.2 (0.12)	27.2 (0.13)	27.4 (0.15)	27.0 (0.24)
Body mass index 25+, *n* (%)	61,384 (69.2)	1255 (70.5)	44 (69.8)	927 (71.0)	630 (71.7)	814 (70.2)	556 (73.4)	181 (68.0)
Cigarettes per day: mean (SE)	10.4 (0.05)	8.9 (0.32)	11.4 (2.04)	8.6 (0.37)	8.5 (0.39)	8.5 (0.46)	8.5 (0.50)	7.9 (0.70)
Ever smoker, *n* (%)	48,080 (50.8)	853 (44.9)	33 (46.5)	610 (43.8)	402 (42.7)	544 (44.0)	355 (43.9)	122 (43.6)
Alcoholic drinks per week: mean (SE)	9.9 (0.04)	10.1 (0.26)	9.9 (1.43)	10.1 (0.30)	9.9 (0.31)	10.0 (0.35)	10.5 (0.40)	9.8 (0.63)
15+ alcoholic drinks per week, *n* (%)	22,369 (24.0)	442 (23.6)	16 (23.2)	329 (23.9)	222 (23.9)	284 (23.2)	194 (24.3)	61 (22.0)
Vigorous physical activity hrs/week: mean (SE)	1.7 (0.01)	1.6 (0.11)	1.6 (0.64)	1.6 (0.13)	1.7 (0.14)	1.8 (0.16)	1.8 (0.18)	1.8 (0.29)
Vigorous physical activity 10+ hrs/week, *n* (%)	2530 (3.2)	61 (4.0)	—	43 (3.9)	33 (4.4)	37 (3.8)	26 (4.1)	11 (5.1)
Moderate physical activity hrs/week: mean (SE)	9.0 (0.04)	10.1 (0.31)	9.1 (1.57)	10.2 (0.36)	10.2 (0.39)	10.1 (0.41)	10.7 (0.51)	10.3 (0.74)
Moderate physical activity 10+ hrs/week, *n* (%)	25,968 (28.4)	632 (34.6)	21 (30.9)	470 (35.1)	318 (35.3)	414 (34.8)	285 (36.6)	106 (38.5)
Family history of prostate cancer, *n* (%)	9693 (10.2)	211 (11.1)	5 (7.0)	158 (11.4)	115 (12.2)	144 (11.6)	78 (9.6)	33 (11.8)
Family history of melanoma, *n* (%)	6746 (7.1)	271 (14.3)	10 (14.1)	198 (14.2)	140 (14.9)	180 (14.6)	118 (14.6)	40 (14.3)
Family history of other cancer, *n* (%)	28,251 (29.8)	626 (33.0)	24 (33.8)	463 (33.3)	306 (32.5)	410 (33.1)	263 (32.5)	90 (32.1)
Non-cancer comorbidities: mean (SE)^b^	0.1 (0.00)	0.3 (0.02)	0.3 (0.12)	0.3 (0.03)	0.3 (0.02)	0.2 (0.02)	0.2 (0.03)	0.2 (0.05)
No non-cancer comorbidities, *n* (%)^b^	89,096 (94.1)	1684 (88.7)	60 (84.5)	1213 (87.1)	853 (90.6)	1101 (89.0)	719 (88.9)	251 (89.6)
GP visits per year > =6, *n* (%)^c^	44,216 (46.7)	1183 (62.3)	56 (78.9)	878 (63.1)	567 (60.2)	767 (62.0)	505 (62.4)	168 (60.0)
PSA screen tests per 5 years > =2, *n* (%)^c^	16,748 (17.7)	370 (19.5)	14 (19.7)	273 (19.6)	191 (20.3)	246 (19.9)	156 (19.3)	61 (21.8)

Percentages shown in the “Total” row represent proportions of the study population. All other percentages represent proportions within melanoma (column) categories excluding missing values.

^a^Characteristic of first melanoma diagnosis between January 1, 1994 and 12 months before baseline.

^b^Non-cancer comorbidities are measured from 5 years prior to baseline date using hospital in-patient data.

^c^Number of GP visits per year and number of PSA-screening tests per 5 years measured from 12 months before baseline to end of follow-up for each participant. All other characteristics measured at baseline.

“—” indicates cell with *n*  < 5 not reported.

**Table 2:** Associations between melanoma diagnosis and subsequent prostate cancer diagnosis.

Melanoma characteristics	Mean age at baseline	Mean # of GP visits/ year^a^	Mean # of PSA screen tests/5 years^a^	PC diagnoses/ total (%)	*p*/years at risk	Std rate^c^	HR_1_ (95% CI)	HR_2_ (95% CI)	HR_3_ (95% CI)	HR_4_ (95% CI)
Melanoma diagnosis										
No	62.0	2.6	1.3	3556/94,649 (4%)	518,059	642	ref.	ref.	ref.	ref.
Yes	67.8	3.2	1.3	121/1899 (6%)	9725	905	1.82 (1.52, 2.18)	1.46 (1.22, 1.75)	1.41 (1.18, 1.70)	1.32 (1.09, 1.60)
* p*-value1							<0.001	<0.001	<0.001	0.005
Number of melanomas										
No melanoma	62.0	2.6	1.3	3556/94,649 (4%)	518,059	642	ref.	ref.	ref.	ref.
1 melanoma	67.6	3.2	1.3	114/1828 (6%)	9388	878	1.77 (1.47, 2.14)	1.43 (1.19, 1.72)	1.38 (1.15, 1.67)	1.28 (1.05, 1.56)
2+ melanomas	71.5	3.8	1.3	7/71 (10%)	337	1449	3.05 (1.45, 6.39)	2.29 (1.09, 4.81)	2.22 (1.05, 4.68)	2.68 (1.20, 5.98)
* p*-value1							<0.001	<0.001	<0.001	0.003
* p*-value2							0.165	0.225	0.227	0.077
Invasiveness of melanoma^b^										
No melanoma	62.0	2.6	1.3	3556/94,649 (4%)	518,059	642	ref.	ref.	ref.	ref.
In situ	68.2	3.2	1.3	34/507 (7%)	2605	998	1.91 (1.36, 2.67)	1.50 (1.07, 2.11)	1.45 (1.04, 2.04)	1.31 (0.92, 1.88)
Invasive	67.6	3.2	1.4	87/1392 (6%)	7120	885	1.78 (1.44, 2.21)	1.44 (1.17, 1.79)	1.40 (1.13, 1.73)	1.32 (1.05, 1.65)
* p*-value1							<0.001	<0.001	<0.001	0.020
* p*-value2							0.740	0.848	0.853	0.986
Breslow thickness of melanoma (mm)^b^										
No melanoma	62.0	2.6	1.3	3556/94,649 (4%)	518,059	642	ref.	ref.	ref.	ref.
0.01–1.00	66.8	3.1	1.4	64/942 (7%)	4882	941	1.91 (1.49, 2.45)	1.57 (1.23, 2.02)	1.50 (1.17, 1.92)	1.43 (1.10, 1.85)
>1.00–2.00	68.1	3.3	1.4	14/214 (7%)	1081	953	1.89 (1.12, 3.19)	1.51 (0.89, 2.55)	1.50 (0.88, 2.54)	1.29 (0.72, 2.32)
>2.00	71.2	3.4	1.2	8/148 (5%)	716	745	1.63 (0.82, 3.27)	1.26 (0.63, 2.52)	1.32 (0.66, 2.65)	1.13 (0.53, 2.42)
Unknown^d^	68.3	3.2	1.3	35/595 (6%)	3046	871	n/a	n/a	n/a	n/a
* p-*value1							<0.001	0.001	0.005	0.047
* p-*value2							0.914	0.834	0.945	0.825
Stage of melanoma^b^										
No melanoma	62.0	2.6	1.3	3556/94,649 (4%)	518,059	642	ref.	ref.	ref.	ref.
In situ	68.2	3.1	1.3	33/499 (7%)	2569	989	1.88 (1.33, 2.64)	1.48 (1.05, 2.08)	1.43 (1.01, 2.02)	1.28 (0.89, 1.85)
Localised	67.5	3.2	1.4	83/1237 (7%)	6386	951	1.90 (1.53, 2.36)	1.54 (1.24, 1.91)	1.49 (1.19, 1.85)	1.39 (1.10, 1.75)
Regional/distant	69.0	3.5	1.2	—	357	318	0.82 (0.20, 3.28)	0.65 (0.16, 2.59)	0.66 (0.16, 2.64)	0.77 (0.19, 3.14)
Unknown^d^	68.6	3.5	1.3	—	412	470	n/a	n/a	n/a	n/a
* p*-value1							<0.001	<0.001	<0.001	0.022
* p*-value2							0.503	0.480	0.521	0.690
Site of melanoma^b^										
No melanoma	62.0	2.6	1.3	3556/94,649 (4%)	518,059	642	ref.	ref.	ref.	ref.
Head and neck	69.5	3.3	1.3	21/412 (5%)	2116	690	1.45 (0.94, 2.22)	1.14 (0.74, 1.74)	1.11 (0.72, 1.70)	1.08 (0.69, 1.69)
Trunk	67.3	3.2	1.3	48/809 (6%)	4131	902	1.70 (1.28, 2.26)	1.37 (1.03, 1.83)	1.34 (1.00, 1.78)	1.22 (0.90, 1.65)
Upper/lower limbs	67.1	3.1	1.4	47/637 (7%)	3269	1030	2.18 (1.66, 2.87)	1.77 (1.34, 2.32)	1.70 (1.29, 2.23)	1.59 (1.18, 2.13)
Unspecified/overlapping^d^	70.2	3.3	1.5	5/41 (12%)	210	2210	n/a	n/a	n/a	n/a
* p*-value1							<0.001	<0.001	<0.001	0.012
* p*-value2							0.219	0.185	0.214	0.277
Time since melanoma diagnosis^b^										
No melanoma	62.0	2.6	1.3	3556/94,649 (4%)	518,059	642	ref.	ref.	ref.	ref.
>1–5 years	67.7	3.2	1.3	65/1040 (6%)	5316	907	1.78 (1.40, 2.28)	1.42 (1.11, 1.82)	1.37 (1.07, 1.75)	1.29 (0.99, 1.67)
>5–10 years	67.6	3.2	1.4	29/579 (5%)	2996	717	1.41 (0.98, 2.04)	1.16 (0.80, 1.67)	1.13 (0.78, 1.63)	1.05 (0.72, 1.54)
>10–15 years	68.3	3.1	1.4	27/280 (10%)	1414	1377	2.80 (1.92, 4.08)	2.25 (1.54, 3.29)	2.18 (1.49, 3.19)	2.05 (1.35, 3.12)
* p*-value1							<0.001	<0.001	<0.001	0.002
* p*-value2							0.034	0.037	0.040	0.058

HR_1_ unadjusted; HR_2_ adjusted for age (as underlying time variable) only; HR_3_ adjusted for age (as underlying time variable), marital status (stratified), private health insurance status, household income, area of residence, education (stratified), BMI, smoking status, alcohol consumption, vigorous physical activity, moderate physical activity, family history of prostate cancer (stratified), family history of melanoma, family history of other cancer, number of non-cancer comorbidities in past 5 years; HR_4_ adjusted for same factors as HR_3_ plus number of GP visits per year (stratified) and number of PSA-screening tests per 5 years (stratified) measured from 12 months before baseline to end of follow-up for each participant.

^a^Mean number of GP visits per year and number of PSA-screening tests per 5 years measured from 12 months before baseline to end of follow-up for each participant.

^b^Characteristic of first melanoma diagnosed between January 1, 1994 and 12 months before baseline.

^c^Rate per 100,000 person-years directly age-standardised to the 2006 NSW male population age distribution.

^d^Unknown or not clearly defined melanoma categories excluded.

“—” indicates cells with *n*  <  5 not reported.

*p*-value1 is for test equality between all non-melanoma and melanoma groups. *p*-value2 is for test equality between melanoma groups only.

During a total follow-up time of 527,784 person-years, 3677 PCs were diagnosed (Table 2). Of the 3677 PC diagnoses, 2037 (55.4%) were localised stage, 388 (10.6%) were regional stage, 81 were distant stage (2.2%) and 1171 (31.8%) were unknown stage. Median follow-up after baseline was 2.9 years among the 3,677 men diagnosed with PC and 5.4 years among the 96,649 men not diagnosed with PC. Six percent of men diagnosed with melanoma (*n* = 1899) during the melanoma diagnosis period were subsequently diagnosed with PC (*n* = 121); 4% men not diagnosed with melanoma (*n* = 94,649) were similarly diagnosed with PC (*n* = 3556). Compared to men not diagnosed with melanoma, those with a melanoma diagnosis were at significantly increased risk of subsequent PC diagnoses (HR_4_ = 1.32; 95% CI [1.09, 1.60]). There was some evidence to suggest that men diagnosed with more than one melanoma during the melanoma diagnosis period might be at higher risk of a subsequent PC diagnoses than men diagnosed with only one melanoma (HR_4_ = 2.68; 95% CI [1.20, 5.98] vs. HR_4_ = 1.28; 95% CI [1.05, 1.56], respectively; *p* = 0.077 for difference between HRs). There was also some evidence that time since first melanoma diagnosis during the melanoma diagnosis period was associated with risk of subsequent PC diagnosis (*p* = 0.058). Specifically, risk of subsequent PC diagnosis was greater for men whose first melanoma diagnosis was >10 to 15 years prior to baseline (HR_4_ = 2.05; 95% CI [1.35, 3.12]) than for men whose melanoma diagnosis was 5 to 10 or 1 to 5 years prior to baseline (HR_4_ = 1.05; 95% CI [0.72, 1.54] and HR_4_ = 1.29; 95% CI [0.99, 1.67], respectively). Risks of PC did not differ according to other melanoma characteristics of invasiveness (*p* = 0.99), Breslow thickness (*p* = 0.83), stage (*p* = 0.69) and site (*p* = 0.28).

Defining the exposure as the most recent melanoma diagnosed in the melanoma diagnosis period rather than the first melanoma in the period, did not appreciably change results (Supplementary Table 2). In another sensitivity analysis, additional adjustment for number of PSA monitoring tests per 5 years did not appreciably change HR estimates (as indicted by the widely overlapping 95% CIs for HR_4_ and HR_5_ in Supplementary Table 3). For example, the “fully adjusted“ HR and 95% CI for “melanoma diagnosis” was HR_4_ = 1.32; 95% CI [1.09, 1.60]) compared to the “over-adjusted” HR of HR_5_ = 1.29; 95% CI [1.06, 1.58]).

## Discussion

Our study is the first to show that the positive association between melanoma and the subsequent risk of PC diagnosis is unlikely to be due to confounding from increased medical surveillance after a melanoma diagnosis. Men with a past melanoma diagnosis were found to be at greater risk of subsequent PC diagnosis after adjusting for frequency of PSA testing and primary healthcare consultations. The positive association remained significant even after the over-adjustment for rate of PSA monitoring tests. The increase in risk of PC remained significant even after the over-adjustment for rate of PSA monitoring tests, and was over 2-fold in men with melanomas diagnosed over 10 years prior and with men diagnosed with multiple melanomas (although this latter finding should be viewed with caution given that it was based on only 7 PC cases from 71 men with more than one melanoma diagnoses)

Our findings are consistent with previous studies. In a recent meta-analysis [11], Acharya et al. pooled the results of 15 (out of 17) studies with 282,592 male melanoma patients and found that men diagnosed with melanoma had a 24% increased risk subsequent PC diagnosis compared to the general male population (standardised incidence ratio (SIR) = 1.24, 95% CI [1.18 to 1.30]). While the meta-analysis identified a moderate amount of heterogeneity between studies (*I*^2^ = 75%), this appears to have primarily been driven by a single study by Wu et al., which reported men diagnosed with melanoma had 73% lower risk of subsequent PC diagnosis compared to the general male population (SIR = 0.270, 95% CI [0.127 to 0.573]) [21]. Apart from a small number of PC cases as their reported outcome (*n* = 8), the authors included ocular melanoma cases in their analysis. The remaining studies in the meta-analysis were relatively homogenous with most showing higher risks of PC diagnoses for melanoma survivors. Similar findings were reported in a 2014 systematic review and meta-analysis of 9 studies (SIR = 1.25, 95% CI [1.13–1.37]), although only three studies were common to both reviews [22]. Notwithstanding the finding of these reviews, all 23 unique studies included in both reviews estimated SIRs using general populations as comparators and, consequently, were unable to control for potential confounding related to medical surveillance.

The relationship between melanoma and PC is neither unidirectional, nor is the subsequent cancer risk specific to PC. The Health Professionals Follow up and Health Physicians studies, each showed that risk of melanoma was increased ~two-fold in PC survivors [23]. A study using the Norwegian cancer registry data, showed that incidence of melanoma was associated with increased risk of PC as well as other cancers, and that overall cancer risk was greater in men than women (RR = 1.51 95% CI [1.41,1.63]) [10]. Analysis of in situ melanoma survivors using data from the Queensland Cancer Registry [9], also showed association with increased risk of PC diagnosis. We found no evidence for increased PC risk in in situ melanoma survivors after adjusting for frequency of PSA testing and GP consultations.

The non-adherence to Hill’s criteria for temporality and specificity, which require the exposure to precede the outcome, as well as for exposure to lead to a single outcome and not multiple outcomes, respectively, does not support Hill’s criteria for causality [24]. A plausible explanation is the presence of a common factor that is associated with both melanoma and PC. Dysregulation of this common factor following the development of the first cancer may increase susceptibility for development of the second cancer, irrespective of which of these cancers are diagnosed first due to dependency of both cancers on this common factor.

The positive association between melanoma and subsequent PC risk, even after adjusting for PSA testing, suggests a paradigm for common risk factors or for cancer-specific correlated risk factors. Androgens are possible candidates as they have long been implicated in prostate carcinogenesis from early developmental androgenic factors such as early onset of pubertal development and severe acne [25–29]. Almost all prostate cancers begin in an androgen-dependent state. Androgens also play an important role in melanogenesis and are known to promote telomere elongation, which is associated with melanoma risk [30, 31]. Analysis of baseline blood collections from the UK Biobank participants showed elevated serum free-testosterone levels in participants who were later diagnosed with melanoma (HR per 50 pmol/L increment = 1.35 *p* = 0.0006) and PC (HR per 50 pmol/L increment = 1.10 *p* = 0.002) [32, 33]. Androgen status may explain some of the gender bias in melanoma survival where male melanoma cases are reported to have poorer survival than female cases, even after adjusting for various lifestyle and behavioural factors [34]. We hypothesise that androgen status in melanoma survivors may have changed in a way that could affect tumour microenvironment and increase cancer susceptibility to androgen-dependent cancers such as prostate cancer, where risk is greater in men with multiple melanomas than only one melanoma.

Research study participants as a group are healthier than the general population and may be more engaged in testing and screening behaviours than the general population, increasing the potential risk of detection bias. Our ability to adjust for rate of PSA testing and monitoring, GP visits and other health comorbidities for each participant through administrative record linkage allowed us to minimise risk of detection bias. However, although we were able to adjust for the important potential confounder “number of PSA-screening tests per 5 years”, the MBS data from which this variable derived from are subject to an important limitation. To limit the cost of Medicare benefits paid for pathology services, Medicare benefits are mostly paid for the three most expensive pathology items in an episode of care only. Consequently, only the three most expensive pathology items in an episode of care are recorded in the MBS data. This administrative practice (commonly referred to as “episode coning”) means that relatively inexpensive pathology tests—such as PSA tests—are under-recorded. A previous study estimated that if all episodes potentially subjected to coning had in fact excluded a PSA test, then about 40% of all PSA tests would not be recorded [35]. A more likely scenario, however, is that the proportion of coned episodes to exclude a PSA test is closer to the proportion of “un-coned” episodes that include a PSA test (where “un-coned” episodes are those that contain two or less pathology items, and sometimes additional items exempt from coning). This scenario would suggest that only about 10% of all PSA tests are not being recorded due to coning. Nonetheless, noting that adjustment for PSA attenuated HRs to some degree, the consequence of coning for our results is that residual confounding from medical surveillance cannot be entirely ruled out. Similarly, although adjusted analyses included age-adjustment, some residual confounding from age remains possible given the age differences between the melanoma groups and the no melanoma group. Findings from the Prostate Cancer data Base Sweden study showed that melanoma risk was highest in men with low-intermediate risk PC and not high-risk disease [36]. It is a limitation that our classification of PC cases does not accurately separate those with clinically significant disease from those with low risk or indolent cancers due to lack of information on clinical pathology.

There are behavioural and molecular factors that may explain the relationship between melanoma incidence and the subsequent increased risk of PC diagnosis. Although our ability to adjust for PC screening reduced risk of detection bias, we acknowledge that residual confounding from increased medical surveillance after melanoma diagnoses cannot be entirely ruled out*.* We hypothesise androgen-dependency as an underlying mechanism that explains the melanoma-PC relationship. A better understanding of the molecular aspects of melanoma in the development of PC may help identify individuals at higher risk for PC.

## Supplementary information

Supplementary Table 1,2,3,4

Reproducibility checklist

## Data Availability

The authors confirm that, for approved reasons, some access restrictions apply to the data underlying the findings. We obtained the data for the project from a third party, namely the Sax Institute, which is the data custodian for the 45 and Up Study. Data are available through application to the Sax Institute. Details are available at https://www.saxinstitute.org.au/our-work/45-up-study/ or through contacting 45andUp.research@saxinstitute.org.au.
