# Supplementary material for: Cutaneous melanoma, prostate-specific antigen testing and the subsequent risk of prostate cancer diagnosis: a prospective analysis of the 45 and Up Study
Source: Br J Cancer. 2022 Nov 1;128(1):71–9. doi: 10.1038/s41416-022-02027-7 (PMC9814593; doi:10.1038/s41416-022-02027-7)
Supplement: Supplementary file 1 — Supplementary Table 1,2,3,4 [file 41416_2022_2027_MOESM1_ESM.docx]

**Supplementary Table 1: Exclusions from the main analysis of all male 45 and Up study participants**

| **Reason for exclusion^:** | **n** | **%** |
| --- | --- | --- |
| **Total 45&up baseline male participants:** | **123, 747** | **100%** |
| Record linkage / data error | 51 | <1% |
| DVA^†^ card holder | 8,739 | 7% |
| Prostate cancer diagnosis before baseline | 7,192 | 6% |
| Other non-melanoma cancer before baseline | 4,197 | 3% |
| Self-reported melanoma before baseline not confirmed by cancer registry | 4,288 | 2% |
| Melanoma diagnosis between 12 months before baseline and end of follow up | 2,732 | 2% |
| **Total excluded participants:** | **27, 341** | **22%** |
| **Total included participants:** | **96,548** | **78%** |

^ Exclusion were applied to participants in descending order of reasons listed in the table. Hence, while a participant may be excluded for more than one reason, only their first listed reason is counted here.

^†^ men who held a Department of Veterans Affairs (DVA) healthcare card

**Supplementary Table 2: Associations between characteristics of most recent melanoma in melanoma diagnosis period and subsequent prostate cancer diagnosis.**

| **Melanoma characteristics** | **Mean age at baseline** | **Mean # of GP visits/ year^** | **Mean # of PSA screen tests/ 5-years^** | **PC diagnoses/ total (%)** | **p/years at risk** | **std rate*** | **HR_1_  (95%CI)** | **HR_2_ (95%CI)** | **HR_3_ (95%CI)** | **HR_4_ (95%CI)** |
| --- | --- | --- | --- | --- | --- | --- | --- | --- | --- | --- |
|  |  |  |  |  |  |  |  |  |  |  |
| **Invasiveness of melanoma^^** | |  |  |  |  |  |  |  |  |  |
| No melanoma | 62.0 | 2.6 | 1.3 | 3556/94649 (4%) | 518059 | 642 | ref. | ref. | ref. | ref. |
| *In situ* | 68.3 | 3.2 | 1.3 | 35/533 (7%) | 2727 | 892 | 1.88 (1.34, 2.62) | 1.47 (1.05, 2.05) | 1.43 (1.02, 1.99) | 1.28 (0.90, 1.82) |
| Invasive | 67.6 | 3.2 | 1.4 | 86/1366 (6%) | 6998 | 911 | 1.79 (1.45, 2.22) | 1.46 (1.18, 1.81) | 1.41 (1.14, 1.75) | 1.33 (1.06, 1.67) |
| *p-value1* |  |  |  |  |  |  | <0.001 | <0.001 | <0.001 | 0.020 |
| *p-value2* |  |  |  |  |  |  | 0.822 | 0.972 | 0.953 | 0.846 |
| **Breslow thickness of melanoma (mm)^^** | | |  |  |  |  |  |  |  |  |
| No melanoma | 62.0 | 2.6 | 1.3 | 3556/94649 (4%) | 518059 | 642 | ref. | ref. | ref. | ref. |
| 0.01-1.00 | 66.8 | 3.1 | 1.4 | 64/924 (7%) | 4793 | 985 | 1.95 (1.52, 2.50) | 1.61 (1.26, 2.06) | 1.53 (1.19, 1.96) | 1.47 (1.13, 1.91) |
| >1.00-2.00 | 68.0 | 3.3 | 1.3 | 14/209 (7%) | 1056 | 978 | 1.93 (1.14, 3.27) | 1.55 (0.92, 2.62) | 1.54 (0.91, 2.61) | 1.31 (0.73, 2.35) |
| >2.00 | 71.1 | 3.4 | 1.2 | 7/143 (5%) | 695 | 615 | 1.47 (0.70, 3.09) | 1.14 (0.54, 2.39) | 1.20 (0.57, 2.51) | 0.99 (0.43, 2.24) |
| Unknown^~^ | 68.4 | 3.2 | 1.3 | 36/623 (6%) | 3180 | 780 | n/a | n/a | n/a | n/a |
| *p-value1* |  |  |  |  |  |  | <0.001 | <0.001 | 0.003 | 0.027 |
| *p-value2* |  |  |  |  |  |  | 0.777 | 0.687 | 0.824 | 0.642 |
| **Stage of melanoma^^** | | |  |  |  |  |  |  |  |  |
| No melanoma | 62.0 | 2.6 | 1.3 | 3556/94649 (4%) | 518059 | 642 | ref. | ref. | ref. | ref. |
| *In situ* | 68.3 | 3.2 | 1.3 | 34/523 (7%) | 2680 | 887 | 1.85 (1.32, 2.60) | 1.45 (1.04, 2.04) | 1.41 (1.00, 1.98) | 1.26 (0.88, 1.80) |
| Localised | 67.4 | 3.1 | 1.4 | 84/1214 (7%) | 6275 | 998 | 1.95 (1.57, 2.43) | 1.59 (1.28, 1.97) | 1.53 (1.23, 1.91) | 1.44 (1.14, 1.81) |
| Regional/distant | 69.1 | 3.5 | 1.2 | - | 357 | 128 | 0.41 (0.06, 2.91) | 0.32 (0.05, 2.30) | 0.33 (0.05, 2.34) | 0.38 (0.05, 2.76) |
| Unknown^~^ | 68.6 | 3.5 | 1.4 | - | 412 | 329 | n/a | n/a | n/a | n/a |
| *p-value1* |  |  |  |  |  |  | <0.001 | <0.001 | <0.001 | 0.008 |
| *p-value2* |  |  |  |  |  |  | 0.296 | 0.269 | 0.293 | 0.371 |
| **Site of melanoma^^** | | |  |  |  |  |  |  |  |  |
| No melanoma | 62.0 | 2.6 | 1.3 | 3556/94649 (4%) | 518059 | 642 | ref. | ref. | ref. | ref. |
| Head and neck | 69.5 | 3.3 | 1.3 | 22/415 (5%) | 2124 | 716 | 1.51 (0.99, 2.30) | 1.18 (0.78, 1.80) | 1.15 (0.76, 1.76) | 1.11 (0.72, 1.72) |
| Trunk | 67.3 | 3.2 | 1.3 | 49/808 (6%) | 4119 | 922 | 1.74 (1.31, 2.30) | 1.41 (1.06, 1.87) | 1.37 (1.03, 1.82) | 1.27 (0.94, 1.70) |
| Upper/lower limbs | 67.0 | 3.1 | 1.4 | 45/634 (7%) | 3265 | 996 | 2.10 (1.59, 2.77) | 1.70 (1.28, 2.24) | 1.63 (1.23, 2.16) | 1.51 (1.12, 2.04) |
| Unspecified/overlapping^~^ | 70.4 | 3.3 | 1.6 | 5/42 (12%) | 216 | 2210 | n/a | n/a | n/a | n/a |
| *p-value1* |  |  |  |  |  |  | <0.001 | <0.001 | <0.001 | 0.022 |
| *p-value2* |  |  |  |  |  |  | 0.393 | 0.339 | 0.379 | 0.479 |
| **Time since melanoma diagnosis^^** | | |  |  |  |  |  |  |  |  |
| No melanoma | 62.0 | 2.6 | 1.3 | 3556/94649 (4%) | 518059 | 642 | ref. | ref. | ref. | ref. |
| >1-5 years | 67.8 | 3.2 | 1.3 | 67/1058 (6%) | 5400 | 914 | 1.81 (1.42, 2.31) | 1.44 (1.13, 1.84) | 1.39 (1.09, 1.77) | 1.29 (1.00, 1.67) |
| >5-10 years | 67.4 | 3.2 | 1.4 | 28/566 (5%) | 2933 | 706 | 1.39 (0.96, 2.02) | 1.14 (0.79, 1.66) | 1.12 (0.77, 1.63) | 1.05 (0.71, 1.55) |
| >10-15 years | 68.3 | 3.1 | 1.4 | 26/275 (9%) | 1392 | 1349 | 2.73 (1.86, 4.02) | 2.20 (1.50, 3.24) | 2.13 (1.44, 3.14) | 2.02 (1.32, 3.10) |
| *p-value1* |  |  |  |  |  |  | <0.001 | <0.001 | <0.001 | 0.003 |
| *p-value2* |  |  |  |  |  |  | 0.044 | 0.049 | 0.055 | 0.075 |

HR_1_ unadjusted; HR_2_ adjusted for age (as underlying time variable) only; HR_3_ adjusted for age (as underlying time variable), marital status (stratified), private health insurance status, household income, area of residence, education (stratified), BMI, smoking status, alcohol consumption, vigorous physical activity, moderate physical activity, family history of prostate cancer (stratified), family history of melanoma, family history of other cancer, number of non-cancer comorbidities in past 5 years; HR_4_ adjusted for same factors as HR_3_ plus number of GP visits per year (stratified) and number of PSA screening tests per 5 years (stratified) measured from 12 months before baseline to end of follow-up for each participant. ^Mean number of GP visits per year and number of PSA screening tests per 5 years measured from 12 months before baseline to end of follow-up for each participant. ^^Characteristic of first melanoma diagnosed between January 1, 1994 and 12 months before baseline. *Rate per 100,000 person-years directly age-standardised to the 2006 NSW male population age distribution. *p-value1* is for test equality between all non-melanoma and melanoma groups. *p-value2* is for test equality between melanoma groups only. ^~^Unknown or not clearly defined melanoma categories excluded.

**Supplementary Table 3:** **Adjusted associations between prostate cancer diagnosis and first melanoma diagnosis 1) “fully-adjusted” models without additional adjustment for number of PSA monitoring tests (HR_4_) and 2) “over-adjusted” models with additional adjustment for number of PSA monitoring tests (HR_5_)**

| **Melanoma characteristics** | **Mean age at baseline** | **Mean # of GP visits/ year^** | **Mean # of PSA screen tests/ 5-years^** | **Mean # of PSA monitor tests/ 5-years^** | **PC diagnoses/ total (%)** | **p/years at risk** | **std rate*** |  | **HR_4_ (95%CI)** | **HR_5_ (95%CI)** |
| --- | --- | --- | --- | --- | --- | --- | --- | --- | --- | --- |
|  |  |  |  |  |  |  |  |  |  |  |
| **Melanoma diagnosis** | |  |  |  |  |  |  |  |  |  |
| No | 62.0 | 2.6 | 1.3 | 1.3 | 3556/94649 (4%) | 518059 | 642 |  | ref. | ref. |
| Yes | 67.8 | 3.2 | 1.3 | 1.7 | 121/1899 (6%) | 9725 | 905 |  | 1.32 (1.09, 1.60) | 1.29 (1.06, 1.58) |
| *p-value1* |  |  |  |  |  |  |  |  | 0.005 | 0.010 |
| **Number of melanomas** | |  |  |  |  |  |  |  |  |  |
| No melanoma | 62.0 | 2.6 | 1.3 | 1.3 | 3556/94649 (4%) | 518059 | 642 |  | ref. | ref. |
| 1 melanoma | 67.6 | 3.2 | 1.3 | 1.6 | 114/1828 (6%) | 9388 | 878 |  | 1.28 (1.05, 1.56) | 1.24 (1.02, 1.52) |
| 2+ melanomas | 71.5 | 3.8 | 1.3 | 2.1 | 7/71 (10%) | 337 | 1449 |  | 2.68 (1.20, 5.98) | 3.33 (1.49, 7.43) |
| *p-value1* |  |  |  |  |  |  |  |  | 0.003 | 0.002 |
| *p-value2* |  |  |  |  |  |  |  |  | 0.077 | 0.019 |
| **Invasiveness of melanoma^^** | |  |  |  |  |  |  |  |  |  |
| No melanoma | 62.0 | 2.6 | 1.3 | 1.3 | 3556/94649 (4%) | 518059 | 642 |  | ref. | ref. |
| *In situ* | 68.2 | 3.2 | 1.3 | 1.7 | 34/507 (7%) | 2605 | 998 |  | 1.31 (0.92, 1.88) | 1.33 (0.93, 1.90) |
| Invasive | 67.6 | 3.2 | 1.4 | 1.6 | 87/1392 (6%) | 7120 | 885 |  | 1.32 (1.05, 1.65) | 1.28 (1.01, 1.62) |
| *p-value1* |  |  |  |  |  |  |  |  | 0.020 | 0.037 |
| *p-value2* |  |  |  |  |  |  |  |  | 0.986 | 0.862 |
| **Breslow thickness of melanoma (mm)^^** | | | |  |  |  |  |  |  |  |
| No melanoma | 62.0 | 2.6 | 1.3 | 1.3 | 3556/94649 (4%) | 518059 | 642 |  | ref. | ref. |
| 0.01-1.00 | 66.8 | 3.1 | 1.4 | 1.6 | 64/942 (7%) | 4882 | 941 |  | 1.43 (1.10, 1.85) | 1.41 (1.08, 1.85) |
| >1.00-2.00 | 68.1 | 3.3 | 1.4 | 1.6 | 14/214 (7%) | 1081 | 953 |  | 1.29 (0.72, 2.32) | 1.33 (0.73, 2.40) |
| >2.00 | 71.2 | 3.4 | 1.2 | 1.7 | 8/148 (5%) | 716 | 745 |  | 1.13 (0.53, 2.42) | 0.92 (0.41, 2.06) |
| Unknown^~^ | 68.3 | 3.2 | 1.3 | 1.7 | 35/595 (6%) | 3046 | 871 |  | n/a | n/a |
| *p-value1* |  |  |  |  |  |  |  |  | 0.047 | 0.064 |
| *p-value2* |  |  |  |  |  |  |  |  | 0.825 | 0.610 |

HR_4_ adjusted for age (as underlying time variable), marital status (stratified), private health insurance status, household income, area of residence, education (stratified), BMI, smoking status, alcohol consumption, vigorous physical activity, moderate physical activity, family history of prostate cancer (stratified), family history of melanoma, family history of other cancer, number of non-cancer comorbidities in past 5 years, number of GP visits per year (stratified) and number of PSA screening and monitoring tests per 5 years (stratified); HR_5_ adjusted for same factors as HR_4_ plus number of PSA monitoring tests per 5 years (stratified). ^ Mean number of GP visits per year and number of PSA screening and monitoring tests per 5 years measured from 12 months before baseline to end of follow-up for each participant. ^^ First melanoma diagnosis between January 1, 1994 and 12 months before baseline. *Rate per 100,000 person-years directly age-standardised to the 2006 NSW male population age distribution. *p-value1* is for test equality between all non-melanoma and melanoma groups. *p-value2* is for test equality between melanoma groups only. ^~^Unknown or not clearly defined melanoma categories excluded.

**Supplementary Table 3 (continued): Adjusted associations between prostate cancer diagnosis and first melanoma diagnosis 1) “fully-adjusted” models without additional adjustment for number of PSA monitoring tests (HR_4_) and 2) “over-adjusted” models with additional adjustment for number of PSA monitoring tests (HR_5_)**

| **Melanoma characteristics** | **Mean age at baseline** | **Mean # of GP visits/ year^** | **Mean # of PSA screen tests/ 5-years^** | **Mean # of PSA monitor tests/ 5-years^** | **PC diagnoses/ total (%)** | **p/years at risk** | **std rate*** | **HR_4_ (95%CI)** | **HR_5_ (95%CI)** |  |
| --- | --- | --- | --- | --- | --- | --- | --- | --- | --- | --- |
|  |  |  |  |  |  |  |  |  |  |  |
| **Stage of melanoma^^** |  |  |  |  |  |  |  |  |  |  |
| No melanoma | 62.0 | 2.6 | 1.3 | 1.3 | 3556/94649 (4%) | 518059 | 642 | ref. | ref. |  |
| *In situ* | 68.2 | 3.1 | 1.3 | 1.7 | 33/499 (7%) | 2569 | 989 | 1.28 (0.89, 1.85) | 1.37 (0.96, 1.97) |  |
| Localised | 67.5 | 3.2 | 1.4 | 1.7 | 83/1237 (7%) | 6386 | 951 | 1.39 (1.10, 1.75) | 1.33 (1.05, 1.69) |  |
| Regional/distant | 69.0 | 3.5 | 1.2 | 1.2 | - | 357 | 318 | 0.77 (0.19, 3.14) | 1.07 (0.26, 4.44) |  |
| Unknown^~^ | 68.6 | 3.5 | 1.3 | 1.5 | - | 412 | 470 | n/a | n/a |  |
| *p-value1* |  |  |  |  |  |  |  | 0.022 | 0.037 |  |
| *p-value2* |  |  |  |  |  |  |  | 0.690 | 0.943 |  |
| **Site of melanoma^^** |  |  |  |  |  |  |  |  |  |  |
| No melanoma | 62.0 | 2.6 | 1.3 | 1.3 | 3556/94649 (4%) | 518059 | 642 | ref. | ref. |  |
| Head and neck | 69.5 | 3.3 | 1.3 | 1.6 | 21/412 (5%) | 2116 | 690 | 1.08 (0.69, 1.69) | 1.06 (0.67, 1.68) |  |
| Trunk | 67.3 | 3.2 | 1.3 | 1.6 | 48/809 (6%) | 4131 | 902 | 1.22 (0.90, 1.65) | 1.17 (0.86, 1.59) |  |
| Upper/lower limbs | 67.1 | 3.1 | 1.4 | 1.7 | 47/637 (7%) | 3269 | 1030 | 1.59 (1.18, 2.13) | 1.59 (1.18, 2.15) |  |
| Unspecified/overlapping^~^ | 70.2 | 3.3 | 1.5 | 1.6 | 5/41 (12%) | 210 | 2210 | n/a | n/a |  |
| *p-value1* |  |  |  |  |  |  |  | 0.012 | 0.017 |  |
| *p-value2* |  |  |  |  |  |  |  | 0.277 | 0.221 |  |
| **Time since melanoma diagnosis^^** | |  |  |  |  |  |  |  |  |  |
| No melanoma | 62.0 | 2.6 | 1.3 | 1.3 | 3556/94649 (4%) | 518059 | 642 | ref. | ref. |  |
| >1-5 years | 67.7 | 3.2 | 1.3 | 1.7 | 65/1040 (6%) | 5316 | 907 | 1.29 (0.99, 1.67) | 1.20 (0.92, 1.56) |  |
| >5-10 years | 67.6 | 3.2 | 1.4 | 1.6 | 29/579 (5%) | 2996 | 717 | 1.05 (0.72, 1.54) | 1.14 (0.77, 1.69) |  |
| >10-15 years | 68.3 | 3.1 | 1.4 | 1.7 | 27/280 (10%) | 1414 | 1377 | 2.05 (1.35, 3.12) | 2.05 (1.33, 3.17) |  |
| *p-value1* |  |  |  |  |  |  |  | 0.002 | 0.006 |  |
| *p-value2* |  |  |  |  |  |  |  | 0.058 | 0.080 |  |

HR_4_ adjusted for age (as underlying time variable), marital status (stratified), private health insurance status, household income, area of residence, education (stratified), BMI, smoking status, alcohol consumption, vigorous physical activity, moderate physical activity, family history of prostate cancer (stratified), family history of melanoma, family history of other cancer, number of non-cancer comorbidities in past 5 years, number of GP visits per year (stratified) and number of PSA screening and monitoring tests per 5 years (stratified); HR_5_ adjusted for same factors as HR_4_ plus number of PSA monitoring tests per 5 years (stratified). ^ Mean number of GP visits per year and number of PSA screening and monitoring tests per 5 years measured from 12 months before baseline to end of follow-up for each participant. ^^ First melanoma diagnosis between January 1, 1994 and 12 months before baseline. *Rate per 100,000 person-years directly age-standardised to the 2006 NSW male population age distribution. *p-value1* is for test equality between all non-melanoma and melanoma groups. *p-value2* is for test equality between melanoma groups only. ^~^Unknown or not clearly defined melanoma categories excluded. – Cell with n<5 not reported.
